# A new generative approach for optical coherence tomography data scarcity: unpaired mutual conversion between scanning presets

**DOI:** 10.1007/s11517-022-02742-6

**Published:** 2023-01-21

**Authors:** Mateo Gende, Joaquim de Moura, Jorge Novo, Manuel G. Penedo, Marcos Ortega

**Affiliations:** 1 Grupo, VARPA, Instituto de Investigación Biomédica de A Coruña (INIBIC), Universidade da Coruña, Xubias de Arriba, 84, A Coruña, 15006 A Coruña Spain; 2 Centro de investigación, CITIC, Universidade da Coruña, Campus de Elviña s/n, A Coruña, 15071 A Coruña Spain

**Keywords:** Optical coherence tomography, Generative adversarial network, Style transfer, Synthetic images

## Abstract

In optical coherence tomography (OCT), there is a trade-off between the scanning time and image quality, leading to a scarcity of high quality data. OCT platforms provide different scanning presets, producing visually distinct images, limiting their compatibility. In this work, a fully automatic methodology for the unpaired visual conversion of the two most prevalent scanning presets is proposed. Using contrastive unpaired translation generative adversarial architectures, low quality images acquired with the faster *Macular Cube* preset can be converted to the visual style of high visibility *Seven Lines* scans and vice-versa. This modifies the visual appearance of the OCT images generated by each preset while preserving natural tissue structure. The quality of original and synthetic generated images was compared using brisque. The synthetic generated images achieved very similar scores to original images of their target preset. The generative models were validated in automatic and expert separability tests. These models demonstrated they were able to replicate the genuine look of the original images. This methodology has the potential to create multi-preset datasets with which to train robust computer-aided diagnosis systems by exposing them to the visual features of different presets they may encounter in real clinical scenarios without having to obtain additional data.

**Graphical Abstract:**
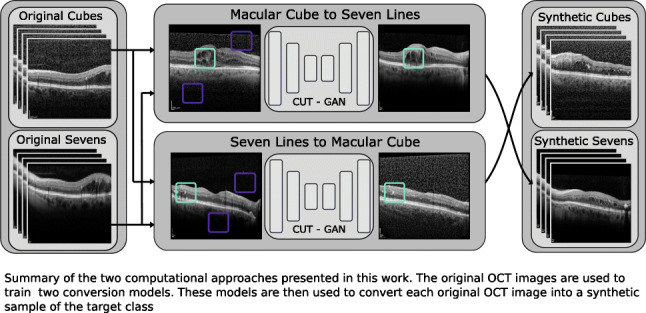
Unpaired mutual conversion between scanning presets. Two generative adversarial models are trained for the conversion of OCT images into images of another scanning preset, replicating the visual features that characterise said preset.

Unpaired mutual conversion between scanning presets. Two generative adversarial models are trained for the conversion of OCT images into images of another scanning preset, replicating the visual features that characterise said preset.

## Introduction

Optical coherence tomography (OCT) is a non-invasive medical imaging technique that can generate cross-sectional, 3-dimensional images of ocular tissue at a micrometre resolution [1]. Thanks to the advances in signal processing, optics and electronics, the quality and resolution of the images obtained through OCT have steadily improved through the years, leading to its widespread use as a diagnostic tool [2], with around 30 million OCT scanning procedures being performed every year worldwide [3]. For reference, OCT imaging has been used to study relevant ocular pathologies such as diabetic macular edema, the most common cause of blindness in patients of diabetes mellitus [4–6]; glaucoma, the leading cause of irreversible blindness worldwide [7–9] and age-related macular degeneration, the leading cause of blindness in people over 50 in the developed world [10–12]; as well as to study the vascular structure of the eye [13–16].

In an OCT scanning session, a low coherence optical beam is swept through the retina of the patient, generating two-dimensional images or B-scans. Due to the forward and backward scattering of light waves, these B-scans present speckle noise, the main quality affecting factor in the OCT images [17]. OCT scanners can be configured to combine and average several B-scans over the same location, leading to a reduction in noise and an overall increase in tissue visibility and quality of details. Nonetheless, this method requires that the tissue does not move throughout the scanning process, to be able to average readings taken at the same point. This requirement limits the overall amount of scans that can be performed in a session due to involuntary eye movements. This translates into a constraint between the total surface of tissue that can be analysed in a given time and the quality of the obtained tomograms.

**Fig. 1:**
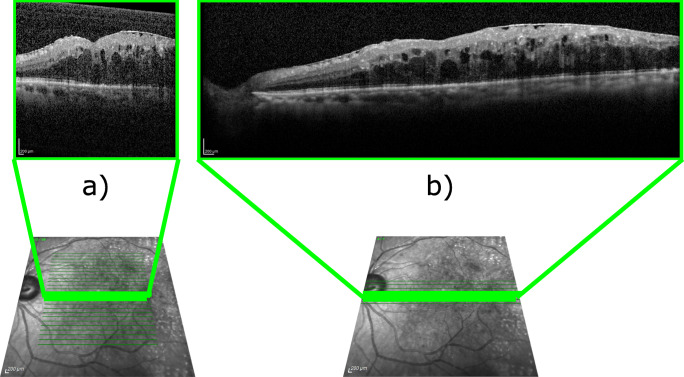
Representative examples of OCT images taken from the same location using the two most common scanning presets. **a** OCT image acquired with the *Macular Cube* preset showing considerable speckle noise. **b** OCT image from the *Seven Lines* preset displaying lower speckle noise and increased tissue visibility

OCT scanner platforms typically provide a series of configuration presets. This way, the specialists can choose which type of scan to be performed according to whether they need to sacrifice quality in order to be able to scan a broader surface of tissue, or whether they can afford the extra time required to perform a higher quality scan. While many presets exist, with different sweeping patterns such as radial or annular scanning, the two most widely used by expert clinicians in medical services are volume scan patterns. The first one scans a square-shaped section of the eye fundus, averaging a small amount of B-scans per final tomogram or image slice. This results in a great number of slices over a wide patch of the retina presenting speckle noise. This scanning preset is known as *Fast Scan* in Heidelberg spectralis$^{{{\circledR }}}$ platforms or *Macular Cube* in Carl Zeiss cirrus-hd$^{{{\circledR }}}$ models, the two most common OCT imaging platforms in clinical settings, and will be referred to as *Macular Cube* in this manuscript. The second one, known as *Seven Lines* in spectralis$^{{{\circledR }}}$ platforms or *Five Line* in cirrus-hd$^{{{\circledR }}}$, is a more intensive scan. In this configuration, a thinner band of the retina is scanned averaging many B-scans per slice. While this scan produces only a few slices over a narrow strip of tissue, these provide much higher visibility, texture detail and image resolution, resulting in images that are much clearer and easier to analyse than the aforementioned preset. We will henceforth refer to this type of scanning preset as *Seven Lines*. An example of the sweeping pattern and a resulting slice for each of these two most representative scanning presets is presented in Fig. 1.

While these presets are the two most prevalent in medical services, this trade-off between scanning time and image quality affects any other configuration, constituting a common paradigm in OCT imaging. Consequently, this leads to a forced choice between the quality of the produced images and the amount of tissue that can be analysed, which therefore results in a shortage of high quality OCT images. Furthermore, the images that are produced by using different presets present visual differences that complicate the development of automatic computer-aided diagnosis (CAD) systems.

Recent years have seen an advancement both in medical imaging techniques as well as in computational architectures and algorithms, leading to the development of new and improved CAD systems based on deep learning [18, 19]. These systems can aid in the detection of several relevant pathologies while achieving equal or better results than board-certified specialists [20–23]. Nonetheless, the development of CAD systems based on machine learning requires well-curated data for training. The images in the training datasets should cover all the possible variabilities in imaging platforms, acquisition conditions, presets and configurations that are present in the clinical setting that the system is intended to work in, so that it can perform its diagnosis functions adequately under such conditions. The labour and economical costs associated with the acquisition of these images, combined with their sensitive nature, lead to an aggravation of the problem of data scarcity, which affects all the domains of application of deep learning [24] and is, therefore, especially prevalent in medical imaging [20].

Given the relevance of this issue, some works have addressed the problem of improving the quality of OCT scans to facilitate the visual inspection of the images and their clinical diagnosis. In 2004, Adler et al. [25] first proposed the use of wavelet filters to reduce the speckle noise in the OCT images. Similarly, other authors continued to improve the image denoising by applying different statistical models such as Bayesian inference [26, 27], non-local means [28] or Huber total variation regularisation [29]. Other approaches expanded upon the wavelet filtering method by applying 3-D block matching techniques [30] or dictionary learning [31]. More recently, some works have approached this task by using deep learning. Apostolopoulos et al. [32] presented a study where they employ an artificial neural network to increase the contrast and reduce the noise in OCT images. Similarly, in the work of Xu et al. [33], a non-linear mapping convolutional neural network is used to perceptually enhance the images resulting in a reduction of the speckle noise. In the work of Seeböck et al. [34], the authors use a generative adversarial network (GAN) to reduce the variability between OCT images acquired with two devices, demonstrating performance gains when using the transformed images for the segmentation of retinal fluid. Lastly, the approach proposed by Huang et al. [35] uses a GAN to both remove the speckle noise that is present in the images and increase the image resolution. These works offer promising results concerning the speckle noise reduction, the perceptual quality enhancement or the increase of resolution of the OCT images, concerning visual inspection. Nevertheless, none of them has addressed the visual differences that exist between images acquired with different scanning presets and their relation to the problem of data scarcity for machine learning-based OCT CAD systems. This leaves the problem of training CAD systems with different presets to be addressed, because while speckle noise can be considered the main quality-affecting factor for OCT images, it is not the only visual difference between images acquired with multiple presets.

To mitigate this problem, this work presents a fully automatic methodology for the mutual unpaired conversion of OCT images that were acquired with different scanning presets. To do this, contrastive unpaired translation architectures are employed for the target conversion. The first approach presented in this work consists of training a model to translate the more numerous images acquired with a low-quality extensive scanning preset such as *Macular Cube* into the style of the higher visibility *Seven Lines* preset to help mitigate the issue of high quality data scarcity in OCT datasets. A second, complementary approach was designed with the intention of performing the opposite translation, transferring the visual features of images obtained with the *Macular Cube* preset to original *Seven Lines* images. The images generated by these two approaches are assessed based on their quality in a complete methodology aimed at determining the optimal point at which the generative models are able to confer the intended visual features of the target preset. This methodology can not only increase the total available number of images by means of oversampling, helping to mitigate the problem of data scarcity that is so prevalent in medical imaging, but it also has the potential to create multi-preset datasets that can be used to train CAD systems in a robust, variability tolerant manner. While this methodology is exemplified through the use of these two most common scanning presets in this work, it is also extensible to any other scanning preset or OCT imaging device as they are all affected by this compromise between scanned area in a given time and image quality. This way, deep learning models can be trained with the visual features of the various presets and acquisition conditions that it may encounter in its use in a clinical environment without the need to procure the otherwise scarce original images acquired with such presets. Preliminary results of this strategy were obtained in [36], demonstrating that this conversion approach can be suitable to address this problem of data scarcity.

## Methods

In this section, the materials and resources that were used for the implementation of this work are covered, along with a description of the OCT image translation methodology. Specifically, the reader can find information on the dataset that was used (Section 2.1), the software and hardware resources (Section 2.2), a description of the image translation methodology (Section 2.3) and an explanation of the experiments conducted to validate the synthetic generated images (Section 2.4).

### Dataset

Regarding the dataset, a total of 1034 OCT images, acquired using a Heidelberg spectralis$^{{{\circledR }}}$ platform, were used. These images were obtained from 56 different patients participating in a study of diabetic macular edema in accordance with the Declaration of Helsinki, approved by the local Ethics Committee of Investigation from A Coruña/Ferrol (2014/437) the 24th of November, 2014. OCT image resolutions ranged from 511 × 495 to 1535 × 495 pixels. In total, 517 of the images were obtained using the *Fast*, or *Macular Cube* preset. In this preset, the scanner sweeps a 20^∘^× 20^∘^ patch of the eye fundus, averaging 9 B-scans to form every tomogram, obtaining 25 slices per scan. The remaining 517 slices were acquired with the *Seven Lines* preset. This preset involves scanning a longer and thinner, 30^∘^× 5^∘^ eye fundus strip and using the average of 25 B-scans for each of the 7 produced tomograms per session. A representative example of the OCT images produced by these presets can be found in Fig. 1.

### Software and hardware resources

In this work, the PyTorch [37] machine learning library (version 1.7.1) under Python (version 3.7.7) for convolutional neural network training and validation was employed. OpenCV [38] (version 3.4.8) and NumPy (version 1.15.0) were used for all image manipulation and processing requirements. Regarding the hardware, the training and validation process of the models was performed on a computer consisting of an NVIDIA GeForce GTX TITAN X GPU, an Intel Xeon E5-2640 CPU and 64 GB of RAM.

### Methodology

To perform the conversion between OCT images, the process was modeled as a “style transfer” approach, in which a neural network attempts to confer the visual features of a target class to an original image while preserving the original structure. In particular, a contrastive unpaired translation generative adversarial network (CUT-GAN) [39] architecture was used for this purpose.

The typical GAN training method involves the use of a generative network *G* and an additional discriminative network *D* whose task is to determine if images belong to the target class from the training set *y* ∈ *Y* or were synthesised by the generative network $\hat {y} = G(x)$, while the generative network trains to maximise the probability that *D* makes a mistake [40]. This way, the training procedure pushes the generator *G* to synthesise images that resemble the target class from the training set, by using an adversarial loss:

1 $$  \mathcal{L}_{\text{GAN}}(X,Y)=\mathbb{E}_{y \sim Y}\log D\left( y\right) + \mathbb{E}_{x \sim X}\log \left( 1-D\left( G\left( x\right)\right)\right) $$

A CUT-GAN architecture is intended for the unpaired translation of images from one domain to another. As such, the generative network *G* consists of an encoder *G*_enc_ and a decoder part *G*_dec_, and its task is to transfer the characterising features of the target domain to original images *x* ∈ *X* without modifying that which is common to both domains. This is achieved by adding a patchwise noise contrastive estimation loss [41] that takes advantage of the ability of the encoder part of the generative network to capture domain-invariant features such as the location of the inner limiting membrane and the choroid, as well as that of the decoder part, which has the means to synthesise domain-specific features like tissue texture as well as speckle noise, or lack thereof.

**Fig. 2:**
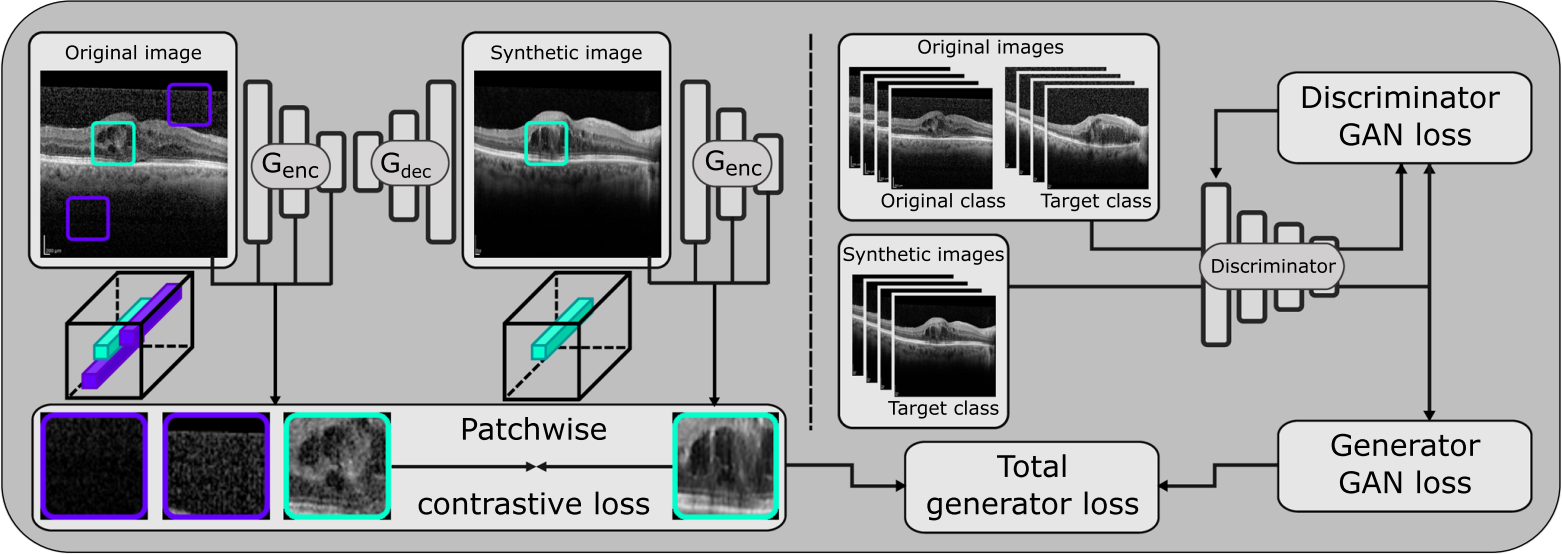
Generative model training procedure: patchwise contrastive loss is calculated by comparing the inner representation of patches that were extracted from both the original and synthetic images. A discriminator is trained in parallel to discern between examples of the original and the target class, which can be used to compute the generator GAN loss

To calculate this contrastive loss, a set of features is extracted from the output from a series of layers *l* ∈{1,2,...,*L*} of the generative encoder. These features are obtained by applying the encoder to patches of both the original $\{\mathbf {z}_{l}\}_{L} = \{H_{l}\left (G^{l}_{\text {enc}}\left (x\right )\right )\}_{L}$ and the synthesised images $\{\hat {\mathbf {z}}_{l}\}_{L} = \{H_{l}\left (G^{l}_{\text {enc}}\left (G\left (x\right )\right )\right )\}_{L}$, passing them through a two-layer MLP network *H*_*l*_. Specifically, a patch is extracted from a location *s* in the original image, along with the a patch extracted from the same location in the synthesised image, and a series of patches from other locations *S* ∖ *s* in the original image. This patchwise loss is then calculated as a cross-entropy loss between the positive and negative examples:

2 $$ \begin{array}{@{}rcl@{}} \mathcal{L}_{\text{Patchwise}}(X) &=& \mathbb{E}_{x\sim X}\sum\limits_{l=1}^{L}{\sum\limits_{s=1}^{S_{l}}{\ell \left( \hat{z}_{l}^{s},{z_{l}^{s}},z_{l}^{S\setminus s}\right)}}\\ \ell(q,q^{+}, q^{-}) &=& -\log \left[ \frac{\exp{\left( q\cdot q^{+}/\tau\right)}}{\exp{\left( q\cdot q^{+}/\tau\right)}+{\sum}_{n=1}^{N}\exp{\left( q\cdot q_{n}^{-}/\tau\right)}}\right], \end{array} $$

where *τ* = 0.07 serves as a temperature with which the distances between the query and the other examples are scaled, and {0,4,8,12,16} are the layers selected for the contrastive loss. By penalising differences in the inner representation of the same image patch in both images, as well as similarities between patches extracted from different regions, the network is trained to preserve the anatomical structure of the eye while changing the visual appearance of the OCT images according to the target preset, as illustrated in Fig. 2.

**Fig. 3:**
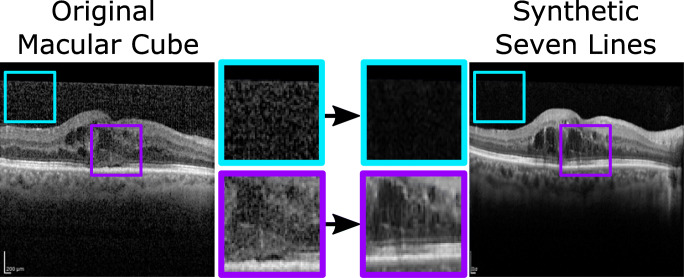
First approach: *Macular Cube* to *Seven Lines* conversion. This conversion produces an overall reduction in the amount of speckle noise over the background and the retina, as well as enhanced tissue texture details in the synthetic generated image

**Fig. 4:**
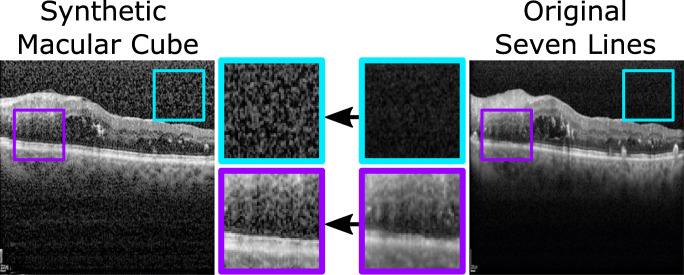
Second approach: *Seven Lines* to *Macular Cube* conversion. In this translation, speckle noise visibility is increased and overall contrast is reduced while preserving the original tissue, producing synthetic generated images that visually resemble original *Macular Cube* scans

In order to address the problem of the visual variability between images acquired with different OCT scanning processes, two complementary approaches were taken, which are detailed below:

1. **First approach:** *Macular Cube* to *Seven Lines* Conversion: The first approach was designed to address the issue of high quality data scarcity in OCT by perceptually increasing the quality of the more numerous *Macular Cube* preset scans. A CUT model was trained to transfer the higher visibility style of *Seven Lines* images to samples acquired with the *Macular Cube* preset, effectively conferring them the reduction in noise of the more intensive scanning preset, along with its more precise visual features. Through this approach, high quality data scarcity can be compensated by converting the more readily available images into the higher quality visual style of *Seven Lines* scans. An example of this conversion is illustrated in Fig. 3.
2. **Second approach:** *Seven Lines* to *Macular Cube* Conversion: The second approach has the complementary purpose of converting higher quality *Seven Lines* scans into the style of the extensive *Macular Cube* preset. Therefore, a second CUT model was trained on the same data to perform the opposite translation. This results in an overall increase in the amount of available images. Moreover, these additional images can be used to train machine learning-based CAD systems in a preset variability-tolerant fashion by exposing them to the different presets that the system may find in a real clinical setting. An example of the visual features of this conversion can be found in Fig. 4.

**Fig. 5:**
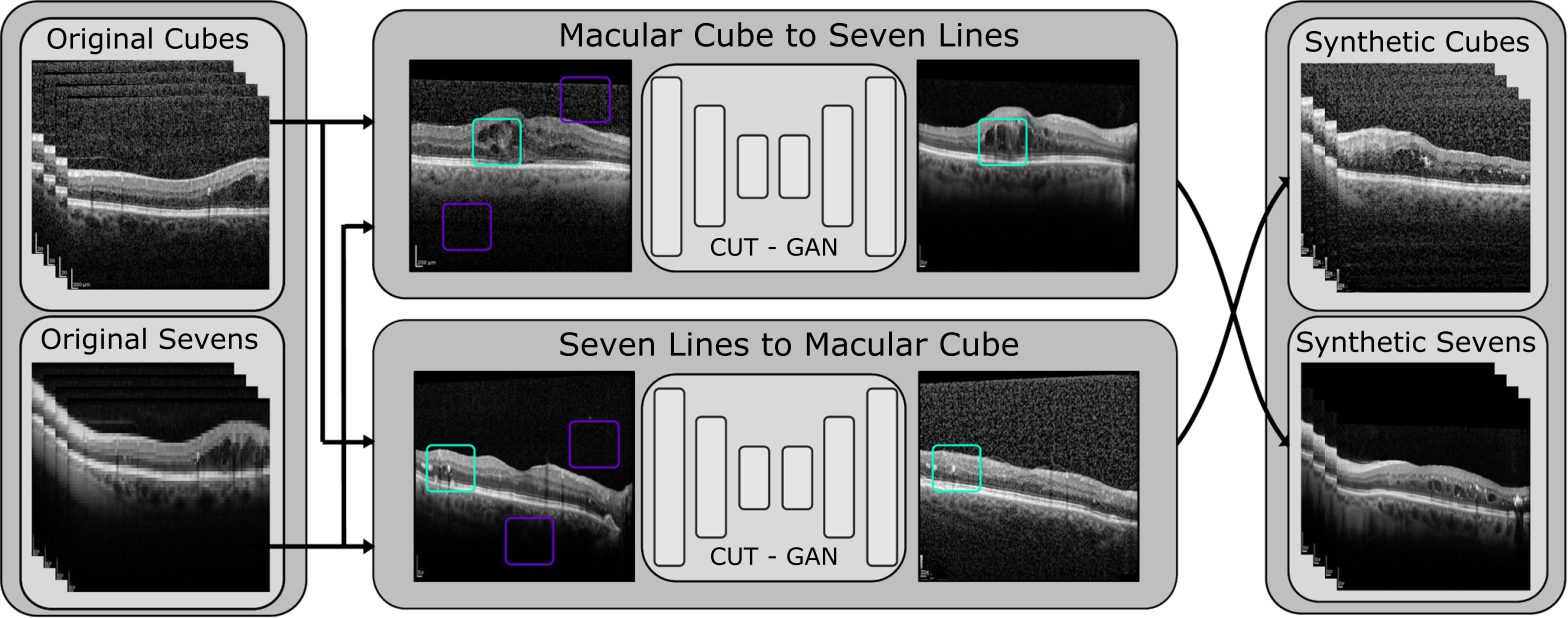
Summary of the two computational approaches presented in this work. The original OCT images are used to train both conversion models. These models are then used to convert each original OCT image into a synthetic sample of the opposite class

For both of these approaches, a residual network [42] backbone with nine residual blocks was used as a base architecture for the CUT generator. The original images were resized to 286 × 286 pixels, with random crops of 256 × 256 being used as training inputs. During the training, the models were optimised using Adam [43] with *β*_1_ = 0.5,*β*_2_ = 0.999 and a learning rate of 2e − 4. The training process lasted for a maximum of 400 epochs, linearly decaying the learning rate for the last 200. Finally, both of these models were used to generate the synthetic counterpart for every original image, as illustrated in Fig. 5.

### Evaluation

A series of experiments were conducted to evaluate the images that were generated by both of the approaches described above. This subsection describes how these experiments were carried out.

In order to validate the perceptual quality of the synthetic images, a qualitative experiment using the Blind/Referenceless Image Spatial Quality Evaluator (brisque) [44] was performed. brisque is an image quality evaluator which, unlike other measures such as Peak Signal to Noise Ratio or Structural Similarity Index Measure [45, 46], does not require a reference image to compare. Instead, it returns a score indicative of the perceptual quality of the processed image, with lower brisque scores indicating a higher image quality. To do this, a series of luminance coefficients are used to measure distortions and their orientations in the image. These are used to compute a series of features at multiple scales. Then, these features are classified and quantified by support vector machines. This way, the different distortions and their effect on image quality and perception can be measured. In this line, brisque has been previously used to assess the quality of medical images with favourable results [47–49]. In this experiment, the brisque score of each set of the original *Macular Cube* and *Seven Lines* images was calculated and compared to those corresponding to the synthetic images.

Complementarily, a set of experiments aimed at measuring the perceptual quality of the synthetic generated images was conducted. The equivalent number of looks (ENL) and the contrast-to-noise ratio (CNR), calculated as $\text {ENL}=\frac {\bar {x}_{\text {BG}}^{2}}{s_{\text {BG}}^{2}}$ $\text {CNR}=\frac {\bar {x}_{\text {ROI}}-\bar {x}_{\text {BG}}}{s_{\text {BG}}}$, where $\bar {x}$ denotes the arithmetic mean and *s* denotes the standard deviation of the intensity values in the images, were used as referenceless image quality estimators. A random representative subset of 100 images of each class was annotated with the location of a homogeneous region of interest (ROI) and the background (BG) containing no tissue. This subset was employed to calculate these estimators. Furthermore, the referenceless Blind Image Quality Index (BIQI) [50] and the Natural Image Quality Evaluator (NIQE) [51] were measured for both the original and synthetic generated images. Section 3.2 covers the results of these experiments.

**Fig. 6:**
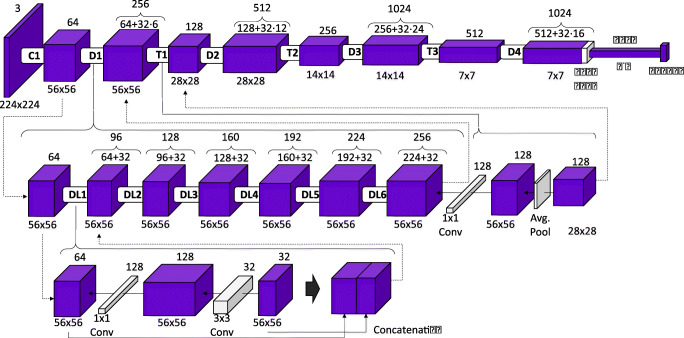
DenseNet-121 model summary. First row shows the complete model. Second row illustrates a dense block structure with its corresponding transition layer. Third row details the composition of every dense layer and the concatenation of inputs and results

Subsequently, the separability of the generated images was assessed. While the training process of a GAN uses a discriminator network *D* to enforce the similarity between original and synthetic images of the target class, this is an intentionally simple architecture in order to avoid overpowering the generator, and it tends to be biased due to the GAN training process. To validate whether the images that were converted between the two presets display the visual features of their target presets, an automatic separability experiment was conducted. In this experiment, an external classifier model was trained to classify between images acquired with the *Macular Cube* preset and images acquired with *Seven Lines*, using a subset of the original images. This network was then tested separately with the remaining original images and with the synthetic generated images. Afterwards, the results produced by the original images were compared with those of the synthetic images. The aim of this experiment is to determine whether the synthetic generated images are classified as their original class or their target class. A densely connected convolutional network [52] architecture was chosen to serve as the external classifier model. This architecture has seen extensive use in medical image classification and screening, surpassing other convolutional neural network architectures [53–56]. Figure 6 illustrates the structure of this model.

Since two separate generative models were trained, one for each approach, each class was tested separately. The Accuracy, calculated as $\text {Accuracy} = \frac {\mathrm {TP + TN}}{\mathrm {TP + TN + FP + FN}}$, was used to evaluate this classification. When testing the two classes separately, positives are considered to be those images of the target class. Due to the absence of true negatives and false positives, the specificity cannot be computed for this test. The results of this experiment can be found in Section 3.3.

Finally, with the purpose to further assess the perceptual validity of these images, a test was conducted to ascertain whether the specialist clinicians are able to discern between the synthetic and original images of both classes. The motivation behind this test was to assess whether these models can preserve the natural tissue structure of the eye, as well as to verify that artificial artefacts are not introduced in the synthetic generated images. The clinicians were asked to classify a random set of 200 images into whether they were acquired with the *Macular Cube* or the *Seven Lines* preset and if they were original images obtained with an OCT platform or they were generated by the network. The results of this experiment are discussed in Section 3.4.

## Results

In this section, the results produced by the aforementioned synthetic image generation methodology are presented, along with those of the tests that were previously described.

### Generative model training

**Fig. 7:**
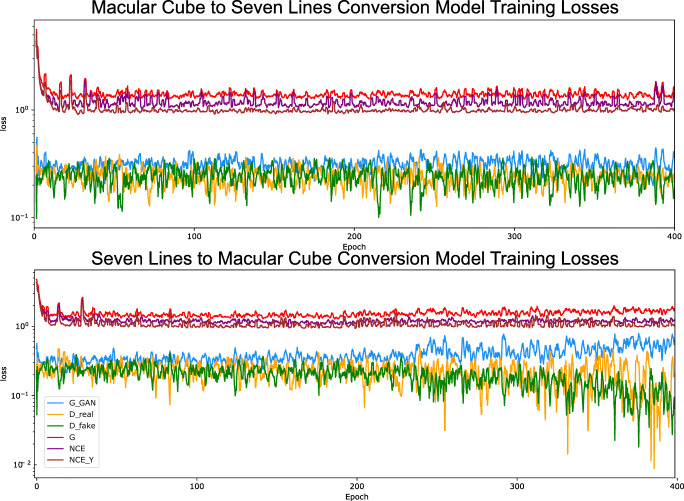
Training losses for both approaches. G_GAN, generator GAN loss; D_real, discriminator loss for real images; D_fake, discriminator loss for synthetic images; G, generator loss; NCE, patchwise loss for images of the original class; NCE_Y, patchwise loss for images of the target class

The curves for both of the GAN losses, along with the contrastive losses for the training of the models are displayed in Fig. 7. These show the loss pattern that is often apparent in GAN training where both discriminator and generator losses tend to converge to a relatively stable value as the training progresses. This mutually dependent stability, however, complicates the task of determining the optimal stopping point of the training process. To work around this problem, both generative models were trained for up to 400 epochs, which was found to be a sufficient length for them to produce satisfactory results. During the training, a checkpoint copy of the generative network state was saved every 20 epochs, for a total of 20 checkpoints per model. At each of these checkpoints, the complete set of images was generated. Inference time at this stage was measured at 250 milliseconds per generated image. Then, the images that were generated by each checkpoint were evaluated using brisque to determine the training checkpoint which produced the best results for each model This process is explained in the next subsection.

### Image quality assessment

**Fig. 8:**
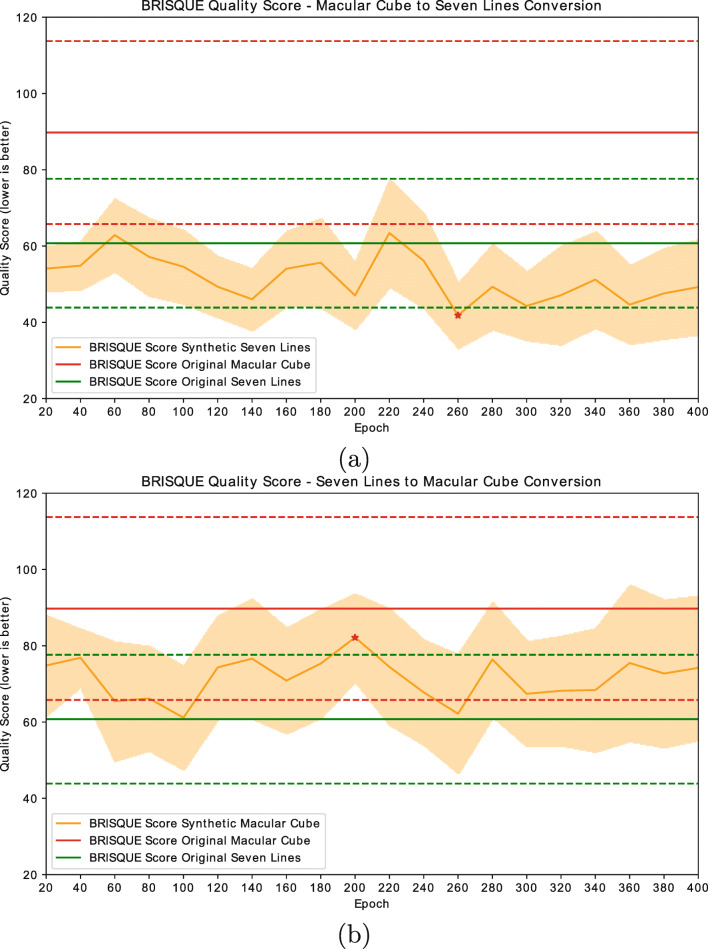
brisque score evolution for each computational approach. **a** 1st approach: Macular Cube to Seven Lines conversion model. **b** 2nd approach: Seven Lines to Macular Cube Conversion model. Red and green solid lines indicate, respectively, the average brisque score for original Macular Cube and Seven Lines images. Dashed lines indicate standard deviation of these sets. A red star indicates the chosen checkpoint for each model

A test was conducted using the brisque score to validate the perceptual quality distribution of the synthetic images. The aim of this experiment was to determine whether the quality distributions of the synthetic generated images are similar to those of the original images. In this experiment, every OCT image for each of the classes in the original dataset was evaluated using brisque. Then, the brisque score for each image generated by every checkpoint of the generative models was calculated and compared to the original images. Figure 8 shows the evolution of the brisque score for both computational approaches for different epochs.

**Fig. 9:**
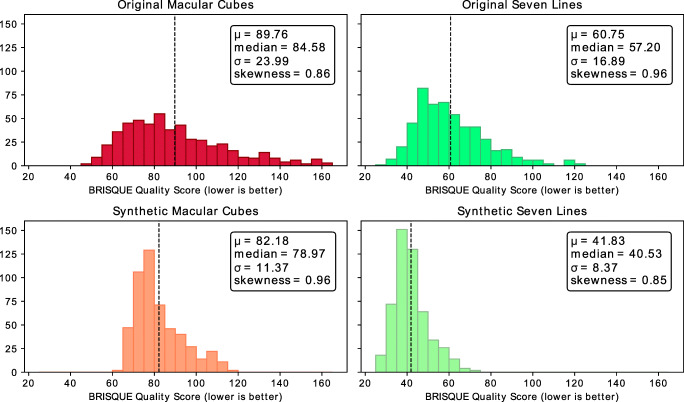
brisque score distribution for each of the data sets, including those generated by the chosen checkpoints of the generative models. *Top left*: Original *Macular Cube* images. *Top right*: Original *Seven Lines* images. *Bottom left*: *Seven Lines* images converted to synthetic *Macular Cube*. *Bottom right*: *Macular Cube* images converted to synthetic *Seven Lines*. Dashed lines indicate the mean value of the distribution

These graphs are indicative of the ability of the generative models to approach the image quality of the target classes. The highest brisque score (corresponding to the lowest perceptual quality) is achieved by the original *Macular Cube* images, represented in red. These images also show the highest variability, with their standard deviation being indicated in red dashed lines. Original *Seven Lines* images, represented in green, show an overall lower brisque score, also having a lower variability than their *Macular Cube* counterparts. Overall, all the synthetic images show a reduced variability in image quality, with synthetic *Seven Lines* images displaying a considerably higher quality than their original *Macular Cube* counterparts and stabilising around the lower fringes of the original *Seven Lines* distribution. In accordance with these results, the lowest scoring of the *Seven Lines*-generating model checkpoints and the highest of the *Macular Cube*-generating model checkpoints were selected, and the brisque score distributions of their generated images were studied. The histograms of the indicated distributions of the original and the synthetic images can be found in Fig. 9.

Complementarily, a representative subset of the original *Macular Cube* images achieved an ENL and a CNR of 94.34 ± 44.41 and 143.35 ± 15.38 respectively, while the synthetic generated macular cube images were measured at 64.81 ± 27.73 and 144.26 ± 10.88. On the other hand, the higher-quality original *Seven Lines* images achieved an ENL and CNR of 152.56 ± 81.08 and 146.88 ± 25.59, while their synthetic counterparts were rated at 188.28 ± 111.08 and 147.68 ± 23.32. In terms of automated image quality scores BIQI and NIQE, the original *Macular Cube* images achieved scores of 25.79 ± 4.20 and 6.42 ± 2.53 respectively, while synthetic generated *Macular Cube* images were rated at 35.62 ± 4.52 and 8.72 ± 1.11. Regarding the *Seven Lines* images, the originals were rated at scores of 24.76 ± 1.84 and 6.05 ± 0.80, while their synthetic counterparts achieved a very similar 24.71 ± 1.84 and 6.12 ± 1.90.

### Separability test

**Fig. 10:**
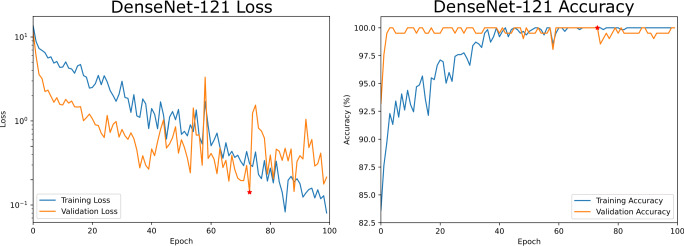
Training and validation losses and accuracies for the DenseNet-121 classifier used to test synthetic image separability. A red star indicates the epoch at which the model produced the lowest validation loss and that was chosen for testing

As previously mentioned, a test was performed to validate the separability of these synthetic images by training a densely connected convolutional network to classify original images between those obtained with the *Macular Cube* preset and those acquired with *Seven Lines*. The original dataset was randomly partitioned in balanced sets, with 60*%* (622 images) forming a training set, 20*%* (206 images) making up a validation set to prevent overfitting and the remaining 206 images being used to test the network. Training and validation losses and accuracy values for this model are presented in Fig. 10.

The synthetic images converted by both of the generative models at the checkpoints that were selected in the previous subsection based on their brisque score were then tested with this network. The accuracy obtained for the original and synthetic dataset is represented in Table 1. It should be clarified that, for the *Macular Cube* to *Seven Lines* model, the positives are the images of the *Seven Lines* class, while the opposite is true for the inverse model.

**Table 1:** Accuracy obtained with the DenseNet-121 model for each image class, both for original and synthetic images

			Accuracy	
		*N*	Macular Cube	Seven Lines
Original images	Training	622	100.00%	100.00%
	Validation	206	100.00%	100.00%
	Test	206	100.00%	100.00%
Synthetic images	Test	1.034	99.42%	100.00%

### Validation by clinical specialists

**Fig. 11:**
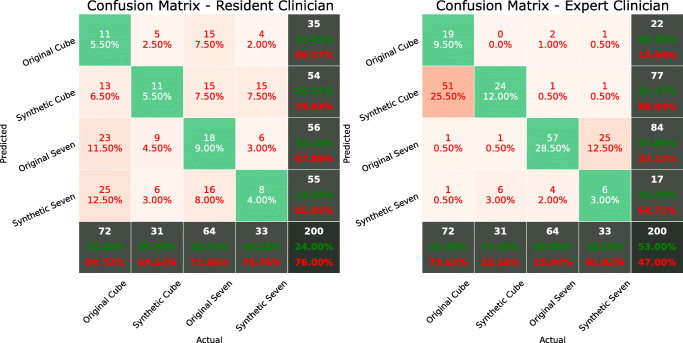
Confusion matrices for the classification results of both specialists

Complementarily, a final test was conducted in order to assess whether medical specialists are able to detect the synthetic images. A random subset of 200 images which are representative of the four classes was created, with 72 of them being original *Macular Cube*, 31 synthetic *Macular Cube*, 64 original *Seven Lines* and 33 synthetic *Seven Lines*. The synthetic images were generated by the models that were selected as described in Section 3.2. Two ophthalmologists from the Hospital Clínico San Carlos in Madrid were asked to determine whether each of the OCT images was of the *Macular Cube* or *Seven Lines* type, and whether they were original or synthetic. One of the clinicians is a medical resident, while the other is an expert specialist with extensive medical experience. The two confusion matrices representing the final results of the test are displayed in Fig. 11.

## Discussion

In this section, the results obtained from the generative methodology as well as those of the tests that were performed to evaluate the synthetic generated images are discussed.

### Image quality assessment

The image quality assessment results obtained from evaluating the synthetic generated images using brisque (Fig. 9) show that all the sets are positively skewed, with most images having a lower brisque score than the mean and a long tail of samples with increasingly higher scores, formed by images with progressively lower quality. This is especially apparent for the original *Macular Cube* samples, which is to be expected of the set with the highest variability in noise and tissue visibility, being the fastest scanning preset considered. Conversely, while the synthetic images also present a similar positive skewness, there is a significant reduction in the amount of unusually highly scored images. These histograms also show the previously mentioned decrease in variability for the synthetic generated images. Overall, the generative models show a great consistency and stability at generating the synthetic images, producing images that show score distributions coherently formed around their respective target quality.

**Fig. 12:**
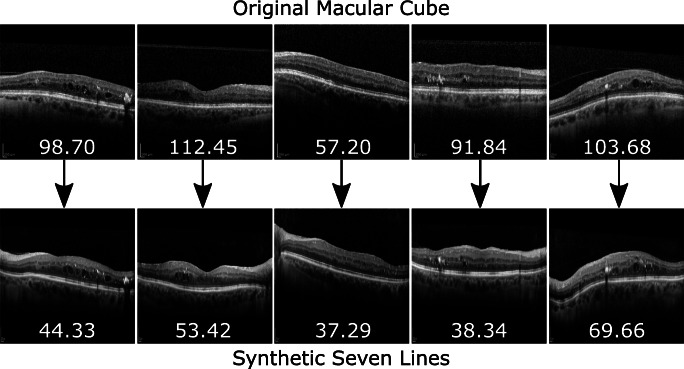
Sample of original *Macular Cube* images and their converted *Seven Lines* synthetic counterparts. Values represent brisque score for each image

**Fig. 13:**
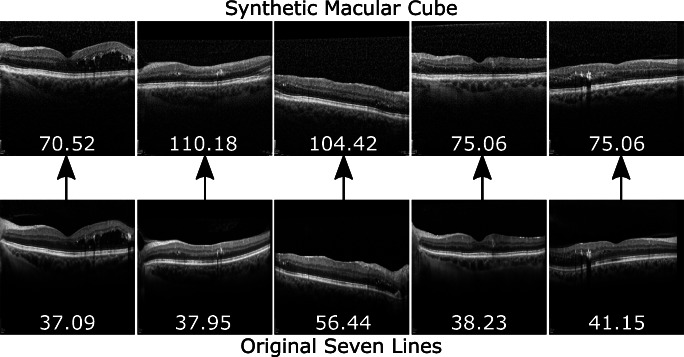
Sample of original *Seven Lines* images and their converted *Macular Cube* synthetic counterparts. Values represent the brisque score for each image

The images were also visually inspected to ensure that the measured changes in brisque score correspond to changes in actual perceptual quality. The synthetic *Seven Lines* images show a significant reduction in speckle noise and tissue visibility in the retina and choroid (Fig. 12). Conversely, regarding synthetic *Macular Cube*, it is apparent that generated images representing the original tissue with an addition of speckle noise and visual features bear resemblance to the original *Macular Cube* samples (see Fig. 13). In both figures, the values of the brisque score correlate with perceptual changes in image visibility, with noisier images being rated higher scores, and images showing greater retinal and choroid visibility achieving lower scores.

**Fig. 14:**
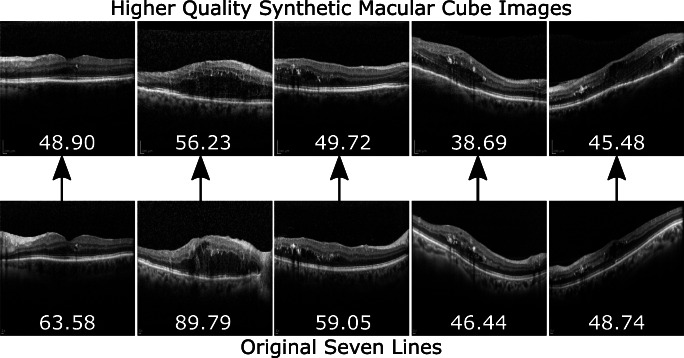
Sample of images generated by a *Seven Lines* to *Macular Cube* model at a point in training in which it actually increases perceptual quality. Values represent the brisque score for each image

Aside from this, the results for the GAN evolution (Fig. 8) show that, for some epochs, synthetic *Macular Cube* images seem to be rated at a similar or higher quality than their original *Seven Lines* counterparts. While this behaviour is not necessarily unusual when training a GAN due to the oscillatory nature of both the discriminator and generative networks, these images were also inspected. A sample of images from training epoch 260 of the *Seven Lines* to *Macular Cube* translating model, in which the network perceptually increased the quality of the *Seven Lines* images, can be found in Fig. 14. This behaviour, combined with the absence of an absolute indicator of GAN training progression, is what motivates the use of an external quality evaluator such as brisque to assess image quality and determine a satisfactory epoch to stop the training process at a point where the generated images present the desired visual features.

### Separability test

The results obtained from the automatic separability test (Table 1) show that the synthetic images are able to mimic the visual features of their target class, with every original *Macular Cube* converted to *Seven Lines* being correctly recognised as a *Seven Lines* image and only 6 synthetic *Macular Cube* images being confused as *Seven Lines*. The synthetic test set contains the images which the network recognises as their original class, but converted to the opposite class. These synthetic generated images were classified by the model as their target class instead of the original one, indicating that they display the intended visual features.

### Validation by clinical specialists

Regarding the ability of the ophthalmology specialists to discern the original and synthetic images of the two presets, the test results (Fig. 11) highlight the difference in experience level between both specialists and its relevance for a complex problem such as this, with the resident achieving an overall accuracy of 24% and the expert correctly guessing more than half of the samples. This complexity is even more evident when evaluating *Macular Cube* and *Seven Lines* separability, with the resident attaining an accuracy of 44% while the expert reached 93%. This indicates that while the OCT images are clearly visually separable according to their acquisition preset, this is by no means a trivial problem, and the ability to do so is acquired with experience. Absence or presence of speckle noise is not enough to differentiate between the images, with other visual features being necessary to distinguish them. When taking into account original and synthetic separability, the results show that the synthetic images are able to deceive even the expert specialist. The expert was not able to determine whether images were originally acquired with a scanner or converted by the generative networks while correctly identifying the visual features that characterise both *Macular Cube* and *Seven Lines* images. Most of the synthetic *Seven Lines* samples, presenting a clearer visibility, were incorrectly classified as original images while, conversely, most of the original *Macular Cube* images, which display more noise and a perceptually worse appearance, were mistakenly identified as synthetic. These results show that the synthetic generated images are effectively indiscernible from the original ones while at the same time preserving the distinctive visual features of their target classes. The obtained results are also indicative of the absence of visual artefacts that could be introduced in the synthetic generated images. Therefore, the tests that were conducted with the specialists demonstrate the substantial performance of the generative models, showing that they are able to generate images that are interchangeable with the original ones even to the expert eye. All of the results obtained indicate that these models are suitable for the purpose of supplying datasets with images converted to the style of different configurations that can be used just as if they were acquired with their target presets.

It should also be highlighted that while other approaches exist in the literature for the denoising or resolution enhancement of low quality images, this proposal is the first to address the problem of data scarcity in OCT through the mutual conversion of images between scanning presets. Due to this focus shift from the enhancement of low quality images to the mutual conversion between visual features within a domain, no comparison with the currently existing methods can be drawn.

## Conclusions

OCT is a relevant medical imaging technique that can be used in conjunction with CAD systems to diagnose relevant ocular pathologies and to study the eye tissue. The overall quality and visibility of the OCT images is considerably affected by light scattering. To overcome this, the OCT scanning platforms typically sample each point multiple times and average the signals to obtain a clearer image. Due to involuntary eye movements, there is a limitation to the amount of samples that can be taken in a scanning session. OCT platforms provide a number of scanning presets that determine the number of scans that are averaged per OCT image, balancing the amount of tissue that is scanned and the quality of the produced images. This compromise between sampled area and image quality leads to a scarcity of high quality data. Moreover, the visual differences that exist between images obtained with different presets limits the potential of datasets based on the scanning preset that was used to acquire their images.

In this work, a complete methodology for the automatic mutual conversion of OCT images has been presented. These OCT images were acquired with the two scanning presets most representative of those used by clinical specialists in medical services: *Macular Cube*, a fast scanning preset which produces 25 eye slices over a square patch of the retina with considerable speckle noise, and *Seven Lines*, an intensive preset that can create cleaner images at the cost of only producing 7 B-scans over a narrow band per session; representing a context of image quality versus quantity compromise that is so widespread in several medical imaging areas. This mutual conversion is achieved by training a contrastive unpaired translation GAN model to translate the more numerous *Macular Cube* images into the higher-visibility style of the intensive *Seven Lines* preset and a second model to perform the complementary conversion. The quality of the synthetic images generated by these models is assessed and compared to the originals in order to determine the optimal training model checkpoint, with an aim to validate the quality of these images and to solve the problem of when to stop the GAN training process.

The experiments that were conducted to validate the synthetic generated images show that these are able to display the visual features of those acquired with their target preset. Qualitatively, the brisque score of original and synthetic images of each preset are very similar, with synthetic images presenting a consistent stability around their target quality distributions. In a validation experiment using a dense convolutional network trained to classify the original images based on their acquisition preset, the synthetic generated images demonstrated a clear separability, being classified as if they were originals of their target preset.

Complementarily, as a way to assess the visual and perceptual qualities of the synthetic images, two ophthalmology specialists with different levels of experience were tasked with classifying images according to whether they are original or synthetic, and according to their acquisition presets. In this experiment, the clinicians were unable to discern between the original and synthetic images, while the expert was clearly able to correctly identify the presets of the originals and the intended target ones of the synthetic generated images. Overall, the generative models demonstrated their ability to provide synthetic generated images that are exceptionally similar to the original ones of their target classes, even to the expert eye.

From the obtained experimental results, it can be concluded that this methodology is able to replicate the visual features of each of the presets in images acquired with another. The synthetic images were validated in terms of perceptual quality, automatic separability and expert separability, with results showing that they resemble their target presets in each of these terms. These generative models can be used to supply OCT datasets limited by their acquisition presets with quality synthetic generated images that display the visual features of any other preset.

Plans for future work include assessing the possible benefits that may be obtained from the paired translation of images in terms of tissue preservation, as well as the possible application of this methodology to produce multi-preset datasets that can be used to train CAD systems in a more robust manner, allowing them to train with all the possible presets it may encounter in a real setting without the need to procure these images. Furthermore, a more elaborate analysis and evaluation of these models and how they perform when trained with images belonging to patients of different ages, sexes and affected by different pathologies is considered for future work. Lastly, this methodology should be considered for the exploration of this context of data scarcity related to image quality and acquisition conditions in other fields of medical imaging where it is so widespread, constituting a paradigm in itself.

## Declarations

This study was performed in line with the principles of the Declaration of Helsinki. Approval was granted by the Ethics Committee of Universidade da Coruña/Ferrol (2014/437) the 24th of November, 2014.
